# Circulating Long Noncoding RNA LNC-EPHA6 Associates with Acute Rejection after Kidney Transplantation

**DOI:** 10.3390/ijms21165616

**Published:** 2020-08-05

**Authors:** Koen E. Groeneweg, Jacques M.G.J. Duijs, Barend W. Florijn, Cees van Kooten, Johan W. de Fijter, Anton Jan van Zonneveld, Marlies E.J. Reinders, Roel Bijkerk

**Affiliations:** Department of Internal Medicine (Nephrology) and the Einthoven Laboratory for Vascular and Regenerative Medicine, Leiden University Medical Center, Albinusdreef 2, 2333 ZA Leiden, Zuid Holland, The Netherlands; k.e.groeneweg@lumc.nl (K.E.G.); J.M.G.J.Duijs@lumc.nl (J.M.G.J.D.); b.w.florijn@lumc.nl (B.W.F.); C.van_Kooten@lumc.nl (C.v.K.); J.W.de_Fijter@lumc.nl (J.W.d.F.); A.J.van_Zonneveld@lumc.nl (A.J.v.Z.); M.E.J.Reinders@lumc.nl (M.E.J.R.)

**Keywords:** long noncoding RNA, kidney transplantation, rejection, microvascular injury

## Abstract

Acute rejection (AR) of a kidney graft in renal transplant recipients is associated with microvascular injury in graft dysfunction and, ultimately, graft failure. Circulating long noncoding RNAs (lncRNAs) may be suitable markers for vascular injury in the context of AR. Here, we first investigated the effect of AR after kidney transplantation on local vascular integrity and demonstrated that the capillary density markedly decreased in AR kidney biopsies compared to pre-transplant biopsies. Subsequently, we assessed the circulating levels of four lncRNAs (LNC-RPS24, LNC-EPHA6, MALAT1, and LIPCAR), that were previously demonstrated to associate with vascular injury in a cohort of kidney recipients with a stable kidney transplant function (*n* = 32) and recipients with AR (*n* = 15). The latter were followed longitudinally six and 12 months after rejection. We found higher levels of circulating LNC-EPHA6 during rejection, compared with renal recipients with a stable kidney function (*p* = 0.017), that normalized one year after AR. In addition, LNC-RPS24, LNC-EPHA6, and LIPCAR levels correlated significantly with the vascular injury marker soluble thrombomodulin. We conclude that AR and microvascular injury are associated with higher levels of circulating LNC-EPHA6, which emphasizes the potential role of lncRNAs as biomarker in the context of AR.

## 1. Introduction

Acute rejection (AR) is considered to be a prominent cause of graft failure in the first year after transplantation in kidney transplant recipients [1,2], although the long-term consequences of AR remain a subject of discussion. Despite better screening and improved immune suppressive therapies, rejection is still suspected to cause a significant proportion of death censored graft failure after kidney transplantation [3,4]. Previous research showed a prolonged effect on kidney function deterioration as well as graft survival after a rejection episode [2]. Microvascular endothelial cells (ECs) are very susceptible to injury, that can result from episodes of AR. Following the alloimmune response, cytokines and growth factors are produced that can lead to EC activation and microvascular destabilization [5,6,7,8,9,10]. These rejection-associated events can result in perpetual EC damage and promotion of (aberrant) angiogenesis within the allograft [5,7,9]. Together, these insults can lead to the loss of the microvasculature, chronic ischemia and cell death [11,12], and ultimately, to the development of interstitial fibrosis/tubular atrophy and graft dysfunction [5,6,9]. Therefore, monitoring the course of microvascular injury after rejection could be beneficial in deciding on the best treatment strategies. Previously, we found the vascular injury markers soluble thrombomodulin (sTM) and Angiopoietin-2 (Ang-2) to increase upon AR. sTM normalized in the first year after AR, while Ang-2 remained elevated [13]. Noncoding RNA, such as micro RNAs (miRNA) and long noncoding RNAs (lncRNA)are increasingly recognized to play an important role in vascular injury [14]. The functions of lncRNAs appear to be very diverse as they can bind DNA, proteins, and other RNAs. E.g. lncRNAs have been demonstrated to serve as a scaffold for transcription factors or can assist chromatin-modifying enzymes, thereby regulating gene expression [15]. LncRNAs were also found to be important for miRNA processing, (alternative) splicing, translation and post-transcriptional regulation, for instance via sponging miRNAs [16,17]. In addition, lncRNAs can be promising biomarkers in a variety of vascular diseases and kidney injury [14,16]. Furthermore, lncRNAs have previously been associated with AR [18], but their dynamics after rejection have not been studied before. Earlier, we described that specific lncRNAs (MALAT1, LNC-RPS24, LNC-EPHA6, and LIPCAR) associate with microvascular damage and angiogenic factors in patients with diabetic nephropathy that received simultaneous kidney-pancreas transplantation [19], but their relation with AR and associated vascular damage is unclear. As such, in this study we first explored the relation of AR with local microvascular injury. Then, in a cross-sectional study of patients with T cell mediated AR, we analyzed selected vascular injury related lncRNAs as potential biomarkers for vascular damage in the context of kidney transplant rejection and assess the dynamics in these lncRNAs after rejection.

## 2. Results

### 2.1. Decreased Capillary Density in Acute Rejection Biopsies

**Figure 1:**
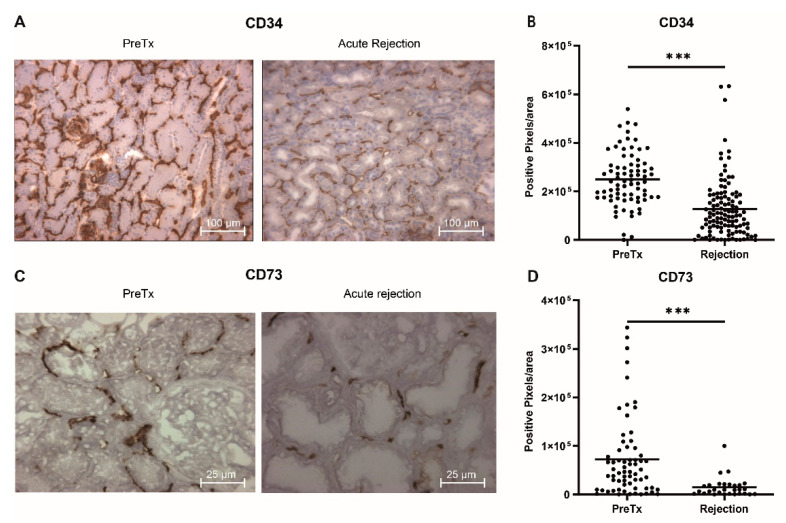
Decreased capillary density after acute rejection. (**A**) Representative images of CD34 staining for pre-transplantation and acute rejection (AR) biopsies. (**B**) Quantification of CD34 staining (PreTx, *n* = 78; AR, *n* = 102). (**C**) Representative images of CD73 staining for pre-transplantation and acute rejection (AR) biopsies. (**D**) Quantification of CD73 staining (PreTx, *n* = 66, AR, *n* = 29). *** *p*-value < 0.001.

To assess the impact of AR on the local capillary density in the kidney, we quantified the number of endothelial cells (EC) and pericytes in archival acute rejection biopsies by immunohistochemical staining of the EC for CD34 antigen and the pericytes for the CD73 marker (resp. *n* = 102 and *n* = 29). Subsequently, we compared these parameters to the available pre-transplant biopsies (resp. *n* = 78 and *n* = 66) of these patients [20]. Patient characteristics can be found in Supplementary Table S1. As shown in Figure 1, we observed a strong decrease in both the number of endothelial cells (~2.5-fold, *p* < 0.0001) as well as pericytes (~6-fold, *p* < 0.0001) in AR, indicating loss of the peritubular capillary network in AR.

### 2.2. Patient Characteristics of Cross Sectional and Longitudinal AR Study Population

Next, we sought to investigate the relation of circulating lncRNAs with AR. To that end, we included plasma samples of a different cross-sectional study cohort that included patients with acute T cell mediated rejection and a control group of patients with stable kidney transplant function after transplantation (hereafter mentioned as ‘stable’). In addition, AR patients were studied longitudinally at 6 and 12 months after rejection to determine the dynamics after AR. The baseline characteristics of the transplant recipients in this cohort are described in Table 1. Most common causes of initial kidney failure before transplantation were autosomal dominant polycystic kidney disease (23%), focal segmental glomerulosclerosis (17%) and IgA nephropathy (13%). The mean time after transplantation (12 months) was comparable. Immunosuppressive regimen did not differ significantly. eGFR was lower and proteinuria higher in patients with AR, compared with stable patients (resp. *p* < 0.001 and *p* = 0.003). Factors that can influence the amount of vascular injury next to rejection, such as donor age, dialysis before transplantation, and months since transplantation, did not differ significantly. Incidence of active smokers was 7% in AR patients and 13% in stable patients. Panel reactive antibodies (PRA), mismatch, immunosuppressive regimen and the presence of previous transplantations did not differ between stable patients and patients with AR. Patients with AR had interstitial rejection, with or without involvement of the vasculature, and were treated with methylprednisolone (67%), ATG alone (13%), or a combination of methylprednisolone and ATG (13%) or alemtuzumab (13%).

### 2.3. Circulating LNC-EPHA6 Levels Directly Correlate with Acute Rejection

**Figure 2:**
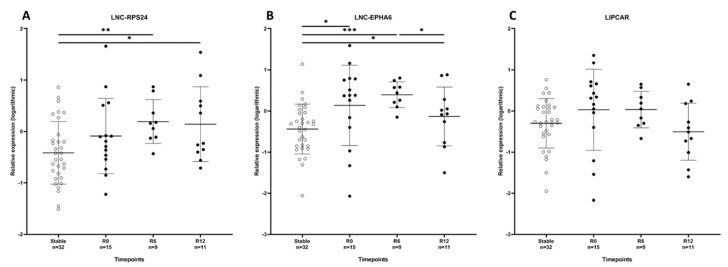
Circulating lncRNA levels are effected by acute rejection. Relative expression of LNC-RPS24 (**A**), LNC-EPHA6 (**B**), and LIPCAR (**C**) in the cross-sectional cohort; kidney recipients with a stable kidney function (Stable; *n* = 32), kidney recipients with acute rejection at the time of rejection (R0), and 6 and 12 months after rejection (R6 and R12). Data are presented as mean ± SD, * *p*-value < 0.05, ** *p*-value < 0.01, *** *p*-value < 0.001.

In order to assess the relationship between AR and vascular injury related lncRNAs LNC-RPS24, MALAT1, LNC-EPHA6, and LIPCAR, circulating levels of these lncRNAs were measured in stable patients and AR patients. In this cohort, MALAT1 levels were only detectable in less than 30% of patients and therefore not included in further analyses. Relative expression of circulating LNC-EPHA6 was significantly higher in patients with AR, compared with stable patients (*p* = 0.017; Figure 2). LNC-RPS24 and LIPCAR showed a similar trend, although these differences did not reach statistical significance (resp. *p* = 0.11 and *p* = 0.16).

### 2.4. Circulating LNC-EPHA6 Decreases in the First Year After Acute Rejection

Since vascular damage persists after a rejection episode, patients with AR were followed longitudinally to study the dynamics of lncRNAs in the first year after AR. Elevated levels of circulating LNC-EPHA6 persisted until six months after AR (*p* < 0.001) and decreased significantly one year after rejection, although LNC-EPHA6 levels at one year after rejection remained slightly higher levels than in stable patients (*p* = 0.03; Figure 2). LIPCAR showed a similar pattern without reaching significance (*p* = 0.16), while LNC-RPS24 increased one year after transplantation (Figure 2). eGFR did not change significantly the year after AR.

### 2.5. LNC-RPS24, LNC-EPHA6 and LIPCAR Correlate with Soluble Thrombomodulin

In order to analyze the association of lncRNAs with vascular injury due to AR, we studied the correlation of LNC-RPS24, LNC-EPHA6, and LIPCAR with vascular injury markers sTM and Ang-2 that were previously assessed [13]. There, we showed a significant increase of sTM levels in patients with acute rejection, followed by a subsequent normalization one year after transplantation, while the ratio between Ang-2 and Ang-1 (mainly determined by Ang-2) significantly increased during AR without significant changes afterwards. Here, no significant associations were found between LNC-RPS24, LNC-EPHA6, and LIPCAR with Ang-2. However, interestingly, LNC-RPS24, LNC-EPHA6, and LIPCAR correlated positively with sTM (Table 2).

## 3. Discussion

Our study shows that levels of circulating LNC-EPHA6 are significantly higher in patients with T cell-mediated AR after renal transplantation, compared with kidney transplant recipients with a stable allograft function. LNC-EPHA6 remains elevated after AR, followed by a decrease one year after rejection. LIPCAR shows a similar pattern, but did not reach statistical significance. In addition, LNC-EPHA6, LIPCAR, and LNC-RPS24 correlate with the vascular injury marker sTM. This suggests that, in particular, LNC-EPHA6 may be related to microvascular damage, of which we confirmed its relation to AR by demonstrating a significantly lower presence of endothelial cells and pericytes in our renal biopsy study.

LNC-EPHA6 was earlier found to relate to diabetic nephropathy [19], but was not studied in the context of AR before. Our finding of higher LNC-EPHA6 levels in patients with AR compared with patients without AR provided proof of principle of the biomarker potential of lncRNAs in AR. However, here we analyzed four pre-selected lncRNAs, thus analyses of other lncRNAs in AR may yield additional associations and may potentially be important for prediction of (vascular injury after) AR. This is in line with two other studies that showed an association between lncRNAs and AR that suggested their value for diagnosis of AR in kidney transplantation [21,22]. Moreover, in a rat study, the lncRNA PRINS was shown to be significantly up-regulated in kidneys of rats with cold ischemia-elicited allograft rejection, compared with rats without rejection [23]. In addition, lncRNAs may also be of value in predicting the development of chronic damage after kidney transplantation [24].

Interestingly, levels of circulating LNC-EPHA6 decrease after AR, while eGFR remains stable. This substantiates that changes in LNC-EPHA6 are likely not to be related to changes in kidney function, but other factors in the pathogenesis of AR, such as persisting microvascular injury. This suggestion is supported by the strong correlation with sTM. However, although significant differences between immunosuppressive drug regimen were not observed, we cannot exclude that differences in rejection treatment altered levels of circulating lncRNAs. Furthermore, an association with the function of the EPHA6 gene might be possible, since lncRNAs are frequently co-regulated and co-expressed with their neighboring genes [25]. The EPHA6 gene is part of a EPH receptor tyrosine kinases family, and thereby interacts with ephrins which subsequently regulates several cellular processes including angiogenesis [26,27].

LIPCAR showed a similar trend after rejection as LNC-EPHA6. This could suggest a similar association as LNC-EPHA6 with rejection. However, changes in LIPCAR did not reach statistical significance due to a large variation. Analysis of LIPCAR in a larger cohort of patients with AR may confirm the link with vascular injury in rejection, since the size of our groups limits the interpretation of LIPCAR in our study. Circulating LNC-RPS24 was only marginally higher in rejection, but increased six months after year after rejection and remained higher. Although speculative, this may be the result of persistent vascular injury after AR or a consequence of the rejection treatment. Lastly, we found LncRNA MALAT1 to be only detectable in less than 30% of the patients in our cohort. Previously, MALAT1 was however detectable in most diabetes mellitus patients [19], suggesting that diabetes mellitus may increase circulating Malat1 levels. However, next to the previously mentioned limited group size, a relatively large spread of lncRNA levels within groups limits the possibility of drawing robust conclusions. The interpretation of subtle changes (e.g., correlation of lncRNAs with the specific Banff classification score for tubulitis, interstitial inflammation, and intimal arteritis) is difficult and larger groups are necessary for the identification of a specific lncRNA as a novel biomarker. However, differences in lncRNAs levels point out the interesting possible added value of lncRNAs in the context of acute cellular rejection. Identification of lncRNAs in the context of antibody-mediated rejection would be interesting as well, since this rare condition also has major implications for the amount of vascular injury.

In conclusion, LNC-EPHA6 is higher in kidney transplant recipients with rejection, compared with those without. This is the first study that shows changes in vascular injury related lncRNAs the first year after rejection. The results suggest that lncRNAs may reflect (micro)vascular damage in the context of rejection and emphasizes the potential role of lncRNAs as biomarkers to monitor vascular injury in kidney transplant rejection.

## 4. Materials and Methods

### 4.1. Renal Biopsy Study

Renal biopsies were selected from patients that had a biopsy proven acute renal allograft rejection, as previously described [20]. Patient and transplantation characteristics are summarized in Supplementary Table S1. Frozen biopsy tissue sections (4 μm) were fixed in acetone, endogenous peroxidase was blocked with H2O2, and slides were blocked with 1% bovine serum albumin and 5% normal human serum in PBS. Sections were then incubated with specific antibodies directed against CD34 (BD Biosciences, Breda, The Netherlands) and CD73 (BD Biosciences, Breda, The Netherlands) followed by appropriate secondary antibodies that were HRP-conjugated (Jackson Immunoresearch, Westgrove, PA, USA). Stainings were visualized using Nova RED (Vector Labs, Peterborough, UK). Quantification of immunohistological staining results was performed using image J software.

### 4.2. Patient Study Cohort

A total of 47 patients were enrolled in a cross-sectional, observational, single center study. All patients were transplanted between 2006 and 2012 in the Leiden University Medical Center (LUMC) in Leiden, The Netherlands. The cohort consisted of 2 groups, namely renal transplant recipients with AR (*n* = 15) and a control group consisting of renal recipients 12 months after transplantation without rejection and with a stable kidney transplant function (*n* = 32). In addition, recipients from the rejection group were followed longitudinally. Plasma samples were obtained at 6 and 12 months after rejection.

The cohort has been described earlier where analysis of circulating Ang-2 and sTM in plasma was performed [13].

All subjects gave their informed consent for inclusion before they participated in the study. The study was conducted in accordance with the Declaration of Helsinki, and the protocol was approved by the Ethics Committee of The Leiden University Medical Center (P09.141).

### 4.3. Immunosuppressive Drugs, Rejection and Rejection Treatment

All patients received immunosuppressive drug therapy according to the standard of care at the time of transplantation. IL-2 receptor inhibitor as induction therapy was the standard of care and alemtuzumab was administered in case the treating physician expected a higher risk of rejection. The presence and type of rejection was assessed using the Banff classification. The choice for a specific rejection treatment was made according to the standard of care at the time of rejection [13].

### 4.4. RNA Isolation

The RNeasy Micro Kit (Qiagen, Venlo, The Netherlands) was used with an adapted protocol, to isolate total RNA from 200 μL plasma. In summary, using 800 trizol μL reagent (Invitrogen, Breda, The Netherlands), the plasma/Trizol sample was centrifuged for 15 min (15,000× g) after the addition of 160 μL chloroform. Then, 100% ethanol (1.5 volume) was added to the aqueous phase and transferred to a MinElute Spin column (Qiagen) followed by centrifugation for 15 s (18,000× g). Subsequently, 700 μL RWT buffer and twice 500 μL RPE buffer was used to wash the column. The column was centrifuged (18,000× g) for 15 s after the first two washing steps and 2 min (18,000× g) after the third washing step. 15 μL RNase-free water was added for elution of the RNA.

### 4.5. RT-qPCR

To quantify circulating lncRNA levels we performed RT-qPCR. Isolated RNA was reverse transcribed using Iscript (Biorad) according to the protocol of the manufacturer. RT-qPCR of target genes was done using SYBR Green Master Mix (Applied Biosystems, Waltham, MA, USA). The primer sequences of target lncRNAs are given in Supplementary Table S2.

### 4.6. Statistical Analyses

Categorical data are described as total count and percentages, parametric data as mean ±standard deviation (SD), and non-parametric data as median and interquartile range (IQR). Testing for differences of baseline characteristics was performed by using Fishers exact test for categorical data and the unpaired *t* test and Mann–Whitney U test for parametric and non-parametric data.

Circulating lncRNA levels were normalized by the double delta CT method to miR-16 and subsequently transformed logarithmically (with base 10). The logarithmic relative expression of all three lncRNAs was normally distributed. In the longitudinal study the data was analyzed by using a linear mixed model analysis. Analysis of correlations between the lncRNAs and vascular markers was performed using Spearman rank correlation.

A *p*-value < 0.05 was considered to be statistically significant. SPSS version 23.0 (SPSS, Inc., Chicago, IL, USA) was used for the data analysis and Graphpad Prism version 8.0 (Graphpad Prism Software, Inc., San Diego, CA, USA).

## Figures and Tables

**Table 1:** Cross-sectional study patient characteristics of patients with a stable kidney transplant function (stable) and patients with acute rejection (AR).

	Stable (*n* = 32)	AR (*n* = 15)	*p*-Value
**Sex, *Male, n*** (**%**)	21 (66%)	10 (67%)	1.00 ^1^
**Age, *Years ± SD***	51 ± 14	54 ± 12	0.35 ^2^
**BMI** (**kg/m^2^**)	26.4 ± 4.6	24.4 ± 3.5	0.15 ^1^
**Preemptive, *n*** (**%**)	16 (50%)	5 (33%)	0.36 ^1^
**Months Since KTx, *Median*** *(**IQR**)*	12 ± 1	12 ± 15	0.97 ^2^
**PRA >5%, *n*** (**%**)	6 (19%)	1 (7%)	0.40 ^1^
**Previous Transplantations, *n*** (**%**)	2 (6%)	3 (20%)	0.31 ^1^
**Mismatch A/B/DR, ** ***Mean***	1.0/1.2/0.8	0.9/1.3/1.0	0.76/0.81/0.63 ^1^
**Donor Characteristics**			
Sex, *male, n (%)*	11 (34%)	7 (47%)	0.52 ^1^
Age, *years ± SD*	50 ± 17	47 ± 12	0.64 ^2^
**Induction Therapy, *n*** (**%**)			0.54 ^1^
Alemtuzumab	3 (9%)	0	
IL-2 receptor inhibitor	29 (91%)	15 (100%)	
**Immunosuppressive Drugs, *n*** (**%**)			
Tacrolimus	22 (69%)	8 (53%)	0.20 ^1^
Cyclosporine	5 (16%)	3 (20%)	1.00 ^1^
Prednisone	32 (100%)	14 (93%)	0.32 ^1^
Mycophenolate mofetil	25 (78%)	8 (53%)	0.07 ^1^
Everolimus	6 (19%)	1 (7%)	0.40 ^1^
**Acute Rejection Therapy, *n*** (**%**)			
ATG		2 (13%)	
methylprednisolone	-	10 (67%)	
methylprednisolone + ATG	-	2 (13%)	
methylprednisolone + alemtuzumab	-	1 (7%)	
**eGFR (mL/min/1.73 m^2^)**	54 ± 12	34 ± 14	<0.001 ^2^
**Proteinuria (g/24 h), Median** *(**IQR**)*	0.17 (0.13–0.25)	0.36 (0.23–1.19)	0.003 ^3^

^1^ Fisher’s exact test, ^2^ unpaired t-test, ^3^ Mann-Whitney U test, KTx = kidney transplantation, PRA = panel reactive antibody.

**Table 2:** Correlation of lncRNAs with vascular injury markers sTM, Ang-2. Values represent correlation coefficient and *p*-value.

	LNC-RPS24	LNC-EPHA6	LIPCAR
Vascular injury markers			
sTM	0.331 (*p* = 0.035)	0.383 (*p* = 0.013)	0.321 (*p* = 0.041)
Ang-2	ns	ns	ns

sTM = soluble thrombomodulin, Ang-2 = Angiopoietin-2.

## Supplementary Materials

Supplementary Materials can be found at https://www.mdpi.com/1422-0067/21/16/5616/s1. Supplementary Table S1: Patient and transplantation characteristics of patients in the renal biopsy study. Supplementary Table S2: Used primer sequences of target lncRNAs.
