# Supplementary material for: Circulating Long Noncoding RNA LNC-EPHA6 Associates with Acute Rejection after Kidney Transplantation
Source: Int J Mol Sci. 2020 Aug 5;21(16):5616. doi: 10.3390/ijms21165616 (PMC7460577; doi:10.3390/ijms21165616)
Supplement: Supplementary file 1 [file ijms-21-05616-s001.pdf]

Supplementary table S1. Patient and transplantation characteristics of patients in the renal biopsy study (n=102)

Variable		Mean ± SD; count (%)
**Recipient**	Age	47.0 ± 12.6
	Age =< 50 years	59 (57.8)
	Age > 50 years	43 (42%)
	Sex (M)	68 (67%)
	Sex (F)	34 (33%)
**donor**	Age	46.2 ± 13.8
	Age =< 50 years	58 (57%)
	Age > 50 years	44 (43%)
	Sex (M)	39 (38%)
	Sex (F)	63 (62%)
**Transplantation type**	Living	25 (25%)
	Post mortal	77 (75%)
**Re-transplantation**		15 (18%)
**Induction therapy**		31 (30%)
**PRA**	0-5%	34 (33%)
	>5%	68 (67%)
**Mismatches**	Class I	
	0-2	78 (80%)
	>2	20 (20%)
	Class II	
	0	32 (33%)
	1-2	65 (67%)
**DGF (need for dialysis)**		29 (28%)
**Need for antibody therapy***		49 (48%)

PRA = panel reactive antibodies, DGF = delayed graft function. *48% of patients with rejection required antibody therapy (antithymocyte globulin) due to a second rejection episode or insufficient response to steroid treatment.

Supplementary table S2. Used primer sequences of lncRNAs.

Primer lncRNA	Sequence
hsa-malat1-fw (MALAT1)	ACCATGGCACTTTCTCCTG
hsa-malat1-rev (MALAT1)	CCCATCACTGAAGCCCACAG
hsa-G003293-fw (LNC-RPS24)	GACGTCGCTATGAACGCTTG
hsa-G003293-rev (LNC-RPS24)	CCAGGTGGGGAGTTTGACTG
hsa-lnc-EPHA6-1:1-fw (LNC-EPHA6)	ATGTTATGCCCGCCTCTTCA
hsa-lnc-EPHA6-1:1-rev (LNC-EPHA6)	TCAGTATTAGAGGCACCGCC
hsa-lipcar-fw (LIPCAR)	TAAAGGATGCGTAGGGATGG
hsa-lipcar-rv (LIPCAR)	TTCATGATCACGCCCTCATA
